# Central nervous system melioidosis: A systematic review of individual participant data of case reports and case series

**DOI:** 10.1371/journal.pntd.0007320

**Published:** 2019-04-25

**Authors:** Monton Wongwandee, Patcharasarn Linasmita

**Affiliations:** Department of Medicine, Faculty of Medicine, Srinakharinwirot University, Nakhon Nayok, Thailand; University of Texas Medical Branch, UNITED STATES

## Abstract

**Background:**

Central nervous system (CNS) melioidosis is rare. However, delayed diagnosis and treatment could lead to fatality. To identify knowledge of CNS melioidosis, we systematically review case reports and case series.

**Methodology/Principal findings:**

We searched through PubMed, Web of Science and Thai-Journal Citation Index databases as well as Google Scholar with the last date on July 10, 2018. The diagnosis of CNS melioidosis had to be confirmed with culture, serology or polymerase chain reaction. We excluded the animal cases and the studies that the clinical data were not available. We identified 1170 relevant studies, while 70 studies with a total of 120 patients were analyzed. Ninety-three percent of patients were reported from the endemic area of melioidosis. Median age was 40 years (IQR 18–53), and 70% were men. A total of 60% had one or more risk factors for melioidosis. The median duration from clinical onset to diagnosis was ten days (IQR 5–25). Fever (82%), headache (54%), unilateral weakness (57%) and cranial nerve deficits (52%) are among the prominent presentation. Most patient (67%) had at least one extraneurological organ involvement. The CSF profile mostly showed mononuclear pleocytosis (64%), high protein (93%) and normal glucose (66%). The rim-enhancing pattern (78%) is the most frequent neuroimaging finding in encephalomyelitis and brain abscess patients. Both brainstem (34%) and frontal lobe (34%) are the most affected locations. Mortality rate was 20%.

**Conclusions/Significance:**

This study is the most extensive systematic review of case reports and case series of CNS melioidosis in all age groups. However, the results should be cautiously interpreted due to the missing data issue. The propensity of brainstem involvement which correlates with prominent cranial nerve deficits is the characteristic of CNS melioidosis especially encephalomyelitis type. The presenting features of fever and neurological deficits (especially cranial nerve palsies) along with the mononuclear CSF pleocytosis in a patient who lives in the endemic area and also has the risk factor for melioidosis should raise the CNS melioidosis as the differential diagnosis.

## Author summary

Melioidosis is an infectious disease caused by the bacterium, *Burkholderia pseudomallei*. It is the disease of the tropical region especially Southeast Asia and northern Australia where the microbes are present in soil and water. Melioidosis can cause a variety of symptoms depending on the site of infection. Brain and spinal cord infections are rare but fatal, and the knowledge of their characteristics is still limited. In this study, the authors found that most patients with central nervous system melioidosis have risk factors especially diabetes mellitus. Fever is the most common presentation, and brainstem is the primary site of attack causing symptoms and signs of cranial nerve abnormalities. Cerebrospinal fluid findings usually show mononuclear cells accompanying with high protein and normal sugar level. The diagnosis is made by isolation of *B*. *pseudomallei* mostly from brain tissue, brain pus or blood. This knowledge would help physicians to recognize the disease which leads to early diagnosis and treatment along with the better outcome.

## Introduction

Melioidosis is an infectious disease caused by the gram-negative bacterium, *Burkholderia pseudomallei*. The regions considered to be endemic include Southeast Asia, northern Australia, South Asia (including India), and China. The disease can involve many organs, but the central nervous system (CNS) melioidosis is rare. Only 1.5 to 5 percent of the melioidosis cases have been reported to have neurological involvement [1,2]. Although there is a systematic review conducted in pediatric patients [3], most CNS melioidosis cases occur in adults. Therefore, the knowledge of the CNS melioidosis including epidemiology, clinical manifestations, laboratory findings, treatment, and outcome are still limited and solely depend on data of case reports. The present study aimed to perform a systematic review of individual participant data of case reports and case series on the CNS melioidosis in all age group.

## Methods

### Search strategy and selection criteria

We conducted a systematic review following the Preferred Reporting Items for a Systematic Review and Meta-analysis of Individual Participant Data (PRISMA-IPD) guideline [4]. However, we did not register our study protocol on the international database of systematic reviews. Inclusion criteria for the study and case recruitment were as follows: 1) the study must be a case report or case series 2) the patient was diagnosed with the CNS melioidosis which includes encephalomyelitis, brain abscess, isolated meningitis and isolated extra-axial collection (defined as subdural empyema or epidural abscess with the absence of encephalomyelitis and brain abscess) 3) the diagnosis of melioidosis was confirmed with either of culture, serology or polymerase chain reaction (PCR) 4) the data of clinical manifestations were available. Exclusion criteria included 1) not case report or case series 2) not the CNS melioidosis case (melioidosis related peripheral nervous system disease, e.g., Guillain-Barré syndrome was also excluded.) 3) animal study 4) no abstract or full text available 5) no clinical data 6) the report language was neither English nor Thai. We searched through bibliographic databases (PubMed, Web of Science, and Thai-Journal Citation Index) and Google Scholar as well as grey literature sources (Bielefeld Academic Search Engine, and Thailand Library Integrated System). The search strategies were as follows: "melioidosis"[mh] AND ("central nervous system infections"[mh] OR meningitis OR "central nervous system" OR neurological OR brain OR cranial OR "spinal cord") for PubMed, TS = melioidosis AND TS = (meningitis OR "central nervous system" OR neurological OR brain OR cranial OR "spinal cord") for Web of Science, "melioidosis" for Thai-Journal Citation Index, and “melioidosis” AND “neurological” AND "case report" for Google Scholar. The filters included human study, and English or Thai language. The last date of searching was on July 10, 2018. The selection process started with firstly, the literature from all sources were gathered and then the studies that duplicated among the databases were excluded. Secondly, abstracts and full texts were screened against the criteria, and individual participant data were sought. The author of the original study was contacted if necessary. The aggregate data was acceptable in case the individual data was eventually not available.

### Data analysis

The collected data on the individual basis included epidemiology, the duration between the onset of illness and the diagnosis, clinical manifestations, cerebrospinal fluid (CSF) profile, imaging findings, diagnostic techniques, types of disease, systemic involvement, treatment, and outcome. The data item that was not available in the study would be recorded as "not reported". Only the reported data would be analyzed. We used the IBM SPSS Statistics for Windows, Version 25.0 for the analysis. Nominal data were reported as frequency and percentage. Scale data were analyzed as mean with SD or median with IQR. Pooled statistics were calculated for analyzing the individual data and aggregate data together. For example, the mean age for all patients was calculated by $\sum \;{\text{n}}_{\text{i}}{\bar{\text{x}}}_{\text{i}}/\sum \;{\text{n}}_{\text{i}}$ where n_i_ was the size of patient group i (individual or aggregate data), and ${\bar{\text{x}}}_{\text{i}}$ was the mean age of patient group i. However, pooled statistics cannot be computed for a median, in case the data is not normally distributed.

### Ethics statement

The authors received approval from the Human Ethics Committee of Srinakharinwirot University to conduct this study (SWUEC/E-369/2560). No written informed consent was required for this systematic review.

## Results

We identified 1170 articles from the bibliographic databases search (PubMed, Web of Science and Thai-Journal Citation Index) including Google Scholar, as well as 82 articles from the grey literature sources. After removal of duplicates, the remaining 1091 records were then assessed for the eligibility through abstracts and full texts. We excluded 1021 studies for particular reasons, and the rest of the 70 studies were sought for the individual participant data. We found 69 studies with 159 subjects for which individual participant data were provided. However, nine individual patients were excluded because the participants duplicate others.

**Fig 1:**
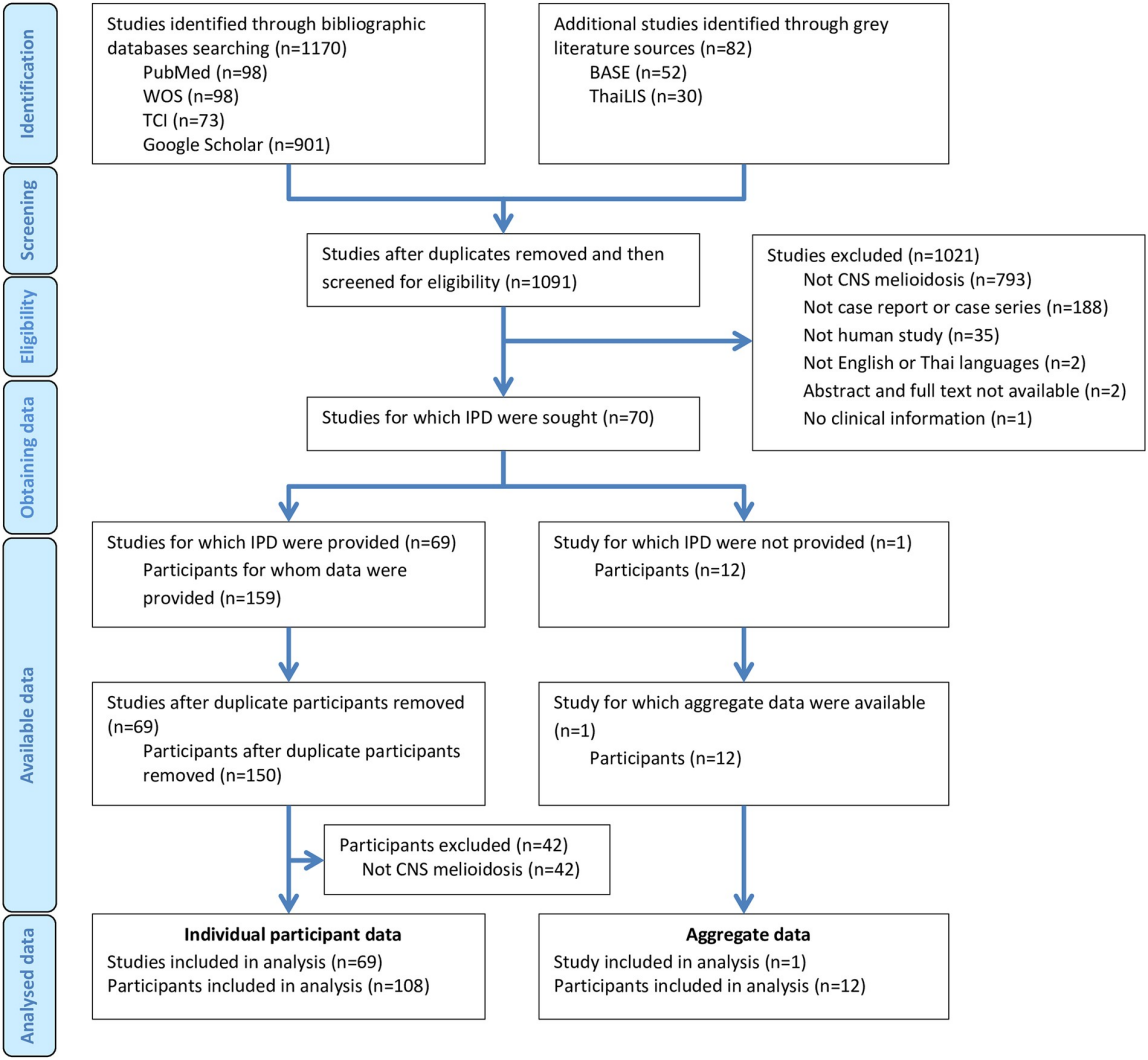
Study selection. WOS, Web of Science; TCI, Thai-Journal Citation Index; BASE, Bielefeld Academic Search Engine; ThaiLIS, Thailand Library Integrated System; IPD, Individual participant data.

Moreover, we excluded the additional 42 cases because the diagnosis was not CNS melioidosis. Finally, we recruited 69 reports including 108 subjects for which individual participant data were provided. For another study that individual participant data were not provided, the aggregate data of 12 participants were available. In total, we recruited 70 studies with 120 patients for the analysis (Fig 1). The first report of a CNS melioidosis patient was presented in 1977. The number of patients per publication ranged from 1 to 12 cases.

**Fig 2:**
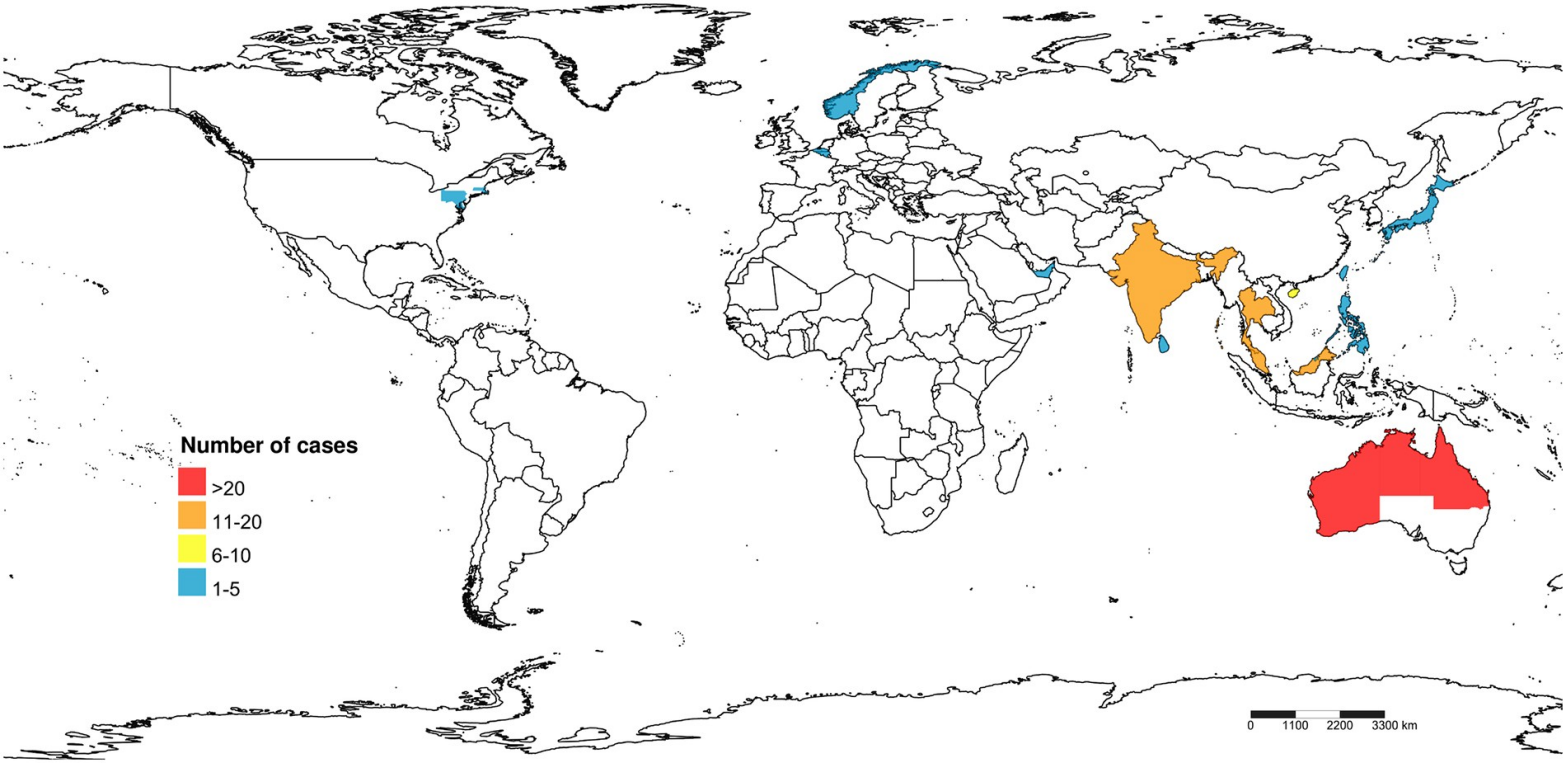
Countries and regions in which the patients of central nervous system melioidosis were reported. The image was created by Shorthouse, David P. 2010. SimpleMappr, an online tool to produce publication-quality point maps, licensed under CC0 1.0 (public domain).

The characteristics of the included studies and individual patients are summarized in the S1 Table. Most cases (93%) were reported from the endemic area of melioidosis in which Australia, Thailand, India, and Malaysia accounted for a majority of patients (Fig 2). Ninety-three percent of Australian patients were reported from the northern part, while 65% of Thai cases were from the northeastern region. Eight cases (7%) were reported from the non-endemic countries including the USA, Belgium, Japan, Norway, and UAE. These patients traveled, lived or served as a soldier in the endemic region at some points of time before the disease development. Fifteen cases worked as rice farmers. Three patients reported the histories of preceding cranial injuries.

The median age of the CNS melioidosis patients was 40 years (IQR 18–53). The 10-day newborn was the youngest reported case, while the oldest was 75 years of age. Most patients (77%) were adults (≥19 years), and 70% of patients were men. Risk factors for melioidosis were shown in Table 1. Most patients (60%) had one or more risk factors. Diabetes mellitus was the most common (43%), but in Australia, excessive alcohol use is the most prominent risk factor (39%). Five cases from Australia were reportedly heavy drinkers of kava, a drink prepared from the powder of the root plant *Piper methysticum*. Contrary to adult patients, most pediatric cases (67%) did not have any risk factors for melioidosis. However, diabetes mellitus remained the only risk factor among children.

**Table 1:** Risk factors for melioidosis, lesion locations, and treatment drugs in the central nervous system melioidosis patients.

	n/N (%)^a^
Risk factor^b^	
Diabetes mellitus	33/76 (43)
Excessive alcohol use	14/63 (22)
Chronic lung disease	1/16 (6)
Chronic kidney disease	2/59 (3)
Kava drinking	5/13 (38)
No risk factor	34/84 (40)
Lesion location^c^	
Frontal lobe	23/68 (34)
Parietal lobe	22/67 (33)
Temporal lobe	9/68 (13)
Occipital lobe	7/67 (10)
Brainstem	22/65 (34)
Cerebellum	15/65 (23)
Treatment drug	
Intensive phase	
Ceftazidime	51/95 (54)
Trimethoprim/ sulfamethoxazole	39/95 (41)
Meropenem	32/91 (36)
Chloramphenicol	12/95 (13)
Imipenem	7/95 (7)
Doxycycline	5/95 (5)
Others	14/95 (15)
Eradication phase	
Trimethoprim/ sulfamethoxazole	54/74 (73)
Doxycycline	20/74 (27)
Amoxicillin/ clavulanic acid	9/74 (12)
Others	5/74 (7)

^a^ n is the number of patients in which the data was present, while N is the total number of patients for which that particular data was reported

^b^ Data for excessive alcohol use, chronic lung disease and chronic kidney disease, which were reported in the publication, were not defined by its author.

^c^ Lesion locations were determined by neuroimaging and presented only for the encephalomyelitis and brain abscess types.

The median duration from clinical onset to diagnosis was ten days (IQR 5–25), and it ranged from 1 to 150 days. Majority of CNS melioidosis patients (91%) was classified as acute melioidosis (less than two months of onset). Encephalomyelitis (37%) and brain abscess (35%) were the two most common disease types. Regarding the age group, encephalomyelitis was most observed in the adult (39%), while brain abscess was the most common type in the children (44%). The cases of encephalomyelitis were reported mainly from Australia (63%). Other types of CNS melioidosis included isolated meningitis and isolated extra-axial collection, which accounted for 16 and 12 percent of cases, respectively. Clinical manifestations of all CNS melioidosis and its types are described in Table 2. Fever was the top presentation for all CNS melioidosis, while other features included headache, altered consciousness, neck stiffness, seizures, unilateral weakness, paraplegia, quadriplegia, and cranial nerve palsies. The facial nerve was the most common cranial nerve affected. In encephalomyelitis patients, the fever and cranial nerve deficits were the prominent manifestations. In brain abscess cases, the most common presentations were the fever and unilateral weakness. Fifteen cases were reported to present with craniofacial swelling. Sixty-seven percent of patients had one or more extraneurological involvement. More than half of those had a pulmonary infection, while one-fourth had localized skin infection (Table 3).

**Table 2:** Clinical manifestations of all CNS melioidosis and its types.

	n/N (%)^a^				
	All CNS melioidosis	Encephalomyelitis	Brain abscess	Isolated meningitis	Isolated extra-axial collection^b^
Fever	93/114 (82)	28/35 (80)	29/39 (74)	15/16 (94)	10/12 (83)
Headache	55/102 (54)	11/30 (37)	24/36 (67)	9/14 (64)	3/10 (30)
Alteration of consciousness	50/97 (52)	14/28 (50)	23/35 (66)	9/13 (69)	3/9 (33)
Neck stiffness	33/55 (60)	9/14 (64)	9/16 (56)	9/11 (82)	0/2 (0)
Seizures	28/106 (26)	6/30 (20)	16/40 (40)	3/14 (21)	3/10 (30)
Unilateral weakness	45/79 (57)	12/21 (57)	25/33 (76)	0/6 (0)	2/7 (29)
Cranial nerve palsies	38/73 (52)	15/23 (65)	13/27 (48)	1/5 (20)	1/6 (17)
CN II	1/67 (1)	1/19 (5)	0/26 (0)	0/5 (0)	0/5 (0)
CN III	8/68 (12)	3/19 (16)	3/26 (12)	1/5 (20)	1/6 (17)
CN IV	5/68 (7)	3/19 (16)	1/26 (4)	0/5 (0)	1/6 (17)
CN V	9/77 (12)	2/23 (9)	1/29 (3)	0/6 (0)	0/7 (0)
CN VI	17/68 (25)	5/19 (26)	6/26 (23)	0/5 (0)	1/6 (17)
CN VII	29/69 (42)	10/21 (48)	11/26 (42)	0/5 (0)	0/5 (0)
CN VIII	0/67 (0)	0/19 (0)	0/26 (0)	0/5 (0)	0/5 (0)
CN IX	16/69 (23)	8/21 (38)	2/26 (8)	0/5 (0)	0/5 (0)
CN X	16/69 (23)	8/21 (38)	2/26 (8)	0/5 (0)	0/5 (0)
CN XI	5/69 (7)	5/21 (24)	0/26 (0)	0/5 (0)	0/5 (0)
CN XII	12/69 (17)	5/21 (24)	1/26 (4)	0/5 (0)	0/5 (0)

CNS, central nervous system

^a^ n is the number of patients in which the data was present, while N is the total number of patients for which that particular data was reported

^b^ Isolated extra-axial collection is defined as subdural empyema or epidural abscess with the absence of encephalomyelitis and brain abscess.

**Table 3:** Extraneurological structure involvements identified in 60 patients of the central nervous system melioidosis.

Structure	Number of cases (%)^a^
Lung	33 (55)
Skin	15 (25)
Spleen	8 (13)
Prostate gland	5 (8)
Liver	4 (7)
Muscle	4 (7)
Eye	4 (7)
Kidney	3 (5)
Joint	2 (3)
Parotid gland	2 (3)
Artery	1 (2)
Bone	1 (2)
Lymph nodes	1 (2)
Pancreas	1 (2)
Tonsil gland	1 (2)

^a^ A patient may have more than one extraneurological organ involved.

Fifty-eight cases underwent lumbar puncture. The CSF profile showed pleocytosis in 91% of the cases, and 64% of patients had CSF mononuclear cell predominance. The CSF WBC count had a median of 190 cells/μL (IQR 54–413), while no CSF RBC was detected in 78% of patients. CSF protein was high (>0.45 g/L) in 93% of cases, and the median protein value was 1.16 g/L (IQR 0.82–1.64). CSF glucose was low (<2.2 mmol/L) in 34% of patients, and the mean glucose level was 2.8 mmol/L (SD 1.5).

Both CT and MRI of the brain were equally done (60% vs. 58% of cases). However, 33% of patients who did the brain CT initially had a normal imaging result, while the brain MRI findings were abnormal in all cases. The imaging findings included hyperintensity on T2W in all MRI cases and also brain edema in 97% of CT and MRI results. There were 49 patients of encephalomyelitis and brain abscess reported having the abnormal contrast enhancement in neuroimaging which the rim-enhancing pattern was the most common characteristic (78%). Other features included nodular (14%), irregular (14%), leptomeningeal (14%) and linear enhancement (2%). Brainstem (34%), frontal lobe (34%) and parietal lobe (33%) were among the most common locations affected in the encephalomyelitis and brain abscess patients (Table 1). Lesions involving contiguous parts of the CNS, which included supra- and infratentorial brain as well as spinal cord, occurs in 29 percent of encephalomyelitis cases. Skull and scalp lesions were present in 19% and 16% of cases, respectively. Twenty-two percent of patients having brain parenchymal diseases also had concurrent involvement of adjacent parts encompassing extra-axial spaces, skull or extracranial structures.

The diagnosis of melioidosis was confirmed with the positive culture in 110 CNS melioidosis patients (92%). Blood was the most common specimen (41%) that grew the organism. Positive cultures of the CNS specimens, which includes brain tissue or pus, CSF and extra-axial collection, were present in 32, 21 and 10 cases, respectively (Table 4). Nine patients (8%) depended on only the serology for diagnosis, which the antibody titer ranged from 1:16 to 1:1,280. There was one case that was diagnosed by only polymerase chain reaction test of blood buffy coat.

**Table 4:** Specimens for which the culture was reportedly positive for *B*. *pseudomallei* in a total of 110 cases of the central nervous system melioidosis.

Specimen	Number of cases (%)^a^
Central nervous system	
Brain tissue or pus	32 (29)
CSF	21 (19)
Extra-axial collection	10 (9)
Blood	45 (41)
Sputum	13 (12)
Skin pus	12 (11)
Scalp pus	6 (5)
Urine	5 (5)
Eye discharge	2 (2)
Parotid gland pus	2 (2)
Bone	1 (1)
Ear discharge	1 (1)
Pleural fluid	1 (1)
Joint fluid	1 (1)
Muscle pus	1 (1)
Skull	1 (1)
Splenic pus	1 (1)

^a^ A patient may have positive cultures on more than one specimen.

Ceftazidime (54% of cases), meropenem (36%) and trimethoprim/sulfamethoxazole (TMP/SMX, 41%) were the primary drugs used for intensive-phase therapy. Others included chloramphenicol (13%), imipenem (7%) and doxycycline (5%) (Table 1). Eight or more weeks of recommended treatment duration was reported in 30 percent of non-death cases. For eradication phase, TMP/SMX was the most common prescription (73%) among others that encompassed doxycycline (27%) and amoxicillin/clavulanic acid (12%). The treatment duration of at least six months was given in 24 percent of non-death patients. Adjunctive abscess drainage was performed in 58 percent of cases. After treatment, 37 percent of the CNS melioidosis patients recovered completely or nearly completely. Thirty-one percent had moderate neurological improvement, while 13 percent did not recover and suffered neurological disability. Overall mortality was 20 percent of patients.

## Discussion

To our knowledge, this is the most extensive systematic review of individual participant data of case reports and case series on the CNS melioidosis. However, the critical issue is that some data were not reported by the author, and we do not know if such variables were absent or not evaluated. This missing data issue makes the data percentage look higher than actual values so the results should be cautiously interpreted.

Like all melioidosis, most CNS melioidosis also occurred in the endemic regions especially northern Australia and Thailand. Some cases developed in the non-endemic region, however, they all had a history of physical presence in the endemic area where they could contact the soil or water before. Because *B*. *pseudomallei* infection can be latent for an extended period, it can then reactivate the disease later like CNS tuberculosis. For example, Beck et al. reported a case of melioidosis meningitis from the US who served during the Vietnam War 13 years ago [5]. This pattern of disease occurrence leads melioidosis to be known as the “Vietnam time bomb”. Some occupations, such as rice farmer, have a potential risk for percutaneous inoculation of the organism via exposure to soils or water.

The pathogenesis of CNS melioidosis appears to be different among the cases. Firstly, hematogenous spreading of the microorganism to the CNS plays an essential role in the disease mechanism. It is supported by the study’s result that blood is the most common specimen that isolates *B*. *pseudomallei*. The circulating bacteria can cross the cellular barriers, which include the blood-brain barrier and blood-CSF barrier, using three methods: transcellular, paracellular or Trojan horse method [6]. The latter mode occurs when the bacteria can survive within the circulating monocytes which can cross the cerebral endothelium via L-selectin (CD62L) mediated migration [7]. Secondly, as evidence from the animal studies, direct brainstem invasion through the nasal passage and cranial nerves is believed to be another distinguish mechanism of CNS melioidosis especially encephalomyelitis type [8]. This mechanism is supported by the results from Darwin study [1] which demonstrated low bacteremic rate for encephalomyelitis in comparison to brain abscess (21% vs. 100%). However, our review has found that the bacteremic rates between cases of encephalomyelitis and brain abscess were quite similar (54% vs. 58%). Finally, percutaneous inoculation over the scalp and subsequent direct extension to the skull and brain may be responsible for the pathophysiology of particular cases. This is supported by the observation that 25 percent of CNS melioidosis patients also had concurrent skull osteomyelitis or scalp abscess, and some indeed reported the history of preceding cranial injury.

All age groups and both sexes could have CNS melioidosis, although most patients were adult males. However, the proportion of children to adult is much higher for the CNS melioidosis (23%) compared to all melioidosis (5%) from the prospective study in northern Australia [9]. CNS melioidosis is a heterogeneous disease that can present as encephalomyelitis, brain abscess, isolated meningitis or isolated extra-axial collection. Most cases of encephalomyelitis were reported from northern Australia, while regional genotypic differences between *B*. *pseudomallei* strains may explain this geographical restriction [10]. Encephalitic syndrome without myelitis became the dominant presentation of encephalomyelitis type. Fever is the most common manifestation, although it also occurs in various infectious diseases. Focal neurological deficits especially cranial nerve palsies are also prominent features of this CNS melioidosis type, in which lesions are mainly located in the brainstem (48%). Sixth, Seventh, Ninth and Tenth cranial nerves are most commonly involved. These cranial nerve deficits can be found in the brainstem encephalitis of other causes as well including *Listeria monocytogenes*, herpes simplex virus type 1, and *Mycobacterium tuberculosis* [11,12,13]. Despite its common brainstem attack, alteration of consciousness develops in only half of cases. Other subtypes of encephalomyelitis include combined encephalitis with myelitis and isolated myelitis. Patients with pure myelitis usually present with classic spinal cord syndrome including paraparesis or quadriparesis accompanying by sensory level and bowel-bladder dysfunction. For brain abscess type, focal neurological deficit, i.e., unilateral weakness is the most common feature; however, cranial nerve palsies occur infrequently. Unlike brain abscess from other causes [14], presentation with fever is more prevalent in melioidosis (74%). Headache is still a common symptom, and most patients have conscious changes. Neck stiffness appears to be associated with occipital lobe abscess. Clinical features of melioidosis meningitis remain similar to other meningitis syndromes which encompass fever, headache, and neck stiffness. Isolated extra-axial collection of melioidosis has not been reported in children so far. Fever remains the most common presentation while headache, focal neurological deficits or seizures is not a reliable indicator of melioidosis subdural empyema or epidural abscess.

Interestingly, CNS melioidosis patients can initially present with cranial swelling, which is associated with infection of extracranial structures such as scalp abscess, parotid abscess or orbital abscess. This manifestation may be the result of direct extension from the intracranial pathology or led by the organism inoculation into structures themselves. Although many organisms other than *B*. *pseudomallei* can cause pneumonia and skin infection, these two organs involved in the CNS melioidosis were not uncommon. Some cases have the splenic abscess, a rare disease which is generally caused by a handful of pathogens including *Klebsiella pneumoniae*, *Escherichia coli*, *Salmonella spp*., and *Staphylococcus aureus* [15]. Moreover, prostatitis or prostatic abscess, which is typically caused by Enterobacteriaceae, was also observed in some cases of CNS melioidosis.

The CSF profile of CNS melioidosis, which commonly shows pleocytosis with mononuclear cells predominance, is comparable to tuberculous or viral meningitis. Nevertheless, tuberculous meningitis typically shows a lower CSF glucose level and higher CSF protein concentration. The CSF polymorphonuclear cells predominance can be demonstrated in one-third of CNS melioidosis patients. This variable characteristic of cells dominance is also shown on the *Listeria* meningoencephalitis [11].

Contrast-enhanced MRI is the neuroimaging study of choice for CNS melioidosis because of its high sensitivity. Brain CT may appear normal if it is done early. Therefore, MRI should be further undergone in case CNS melioidosis is still in the differential diagnosis. Brainstem lesions are seen to be prominent in the encephalomyelitis type. Some authors suggested that CNS melioidosis shows a propensity to involve and spread along the white matter tracts across the commissural or longitudinal fibers [16,17]. This may explain the study’s result that a reasonable proportion of encephalomyelitis patients had lesions involving both supra- and infratentorial structures as well as the spinal cord. However, isolated supratentorial lesions are found to be the most common findings of all CNS melioidosis and even all encephalomyelitis types. Interestingly, some parenchymal lesions also involve adjacent subdural or epidural spaces, skull and extracranial structures. This invasive behavior is similar to tuberculosis, invasive fungal infection and malignant neoplasms.

Culture from the CNS specimen is the best way to establish a definite diagnosis of CNS melioidosis. These sources encompass 1) brain tissue 2) pus from the brain or subdural collection or epidural abscess and 3) CSF. However, it is hard to obtain the specimen from some pathogenic location such as the brainstem. As a result, the diagnosis can be made by the presentation of the compatible CNS diseases that is supported by positive cultures from other specimens. The hemoculture yields a positive result in the highest sensitivity with 52 percent. Other samples including sputum, skin pus, osteomyelitic skull, and scalp abscess would be helpful for the diagnosis. Although we included 9 CNS melioidosis cases who were diagnosed by only serology in our review, the indirect hemagglutinin assay (IHA) alone is not reliable enough for diagnosis in clinical practice because of its problematic false positive and false negative [18,19].

Once the diagnosis of CNS melioidosis was made, most cases were treated with appropriate drugs both in the intensive- and eradication-phase therapies. According to the 2010 consensus recommendations, ceftazidime and meropenem are the drugs of choice for intensive-phase therapy, while trimethoprim/sulfamethoxazole is the first-line drug for eradication-phase therapy [20]. Some medications including chloramphenicol and doxycycline were used in intensive-phase therapy in some CNS melioidosis cases; however, they are not effective thus should be avoided. The treatment duration is also a key to success that eight weeks and six months are the minimal duration for intensive- and eradication-phase therapy, respectively [21]. However, we found that most non-death cases were treated inadequately. Although most cases of CNS melioidosis completely or partially recovered following treatment, one-fifth finally succumbed to the disease.

There are several limitations to our study besides the missing data issue. We identified only the CNS melioidosis cases that were presented in the literature. However, we believe that the CNS melioidosis patients were still underreported especially in the endemic regions of melioidosis. The precise diagnosis of CNS melioidosis types which differentiates encephalomyelitis from brain abscess was not definite. Some large rim-enhancing lesions diagnosed with brain abscess might be progressed from prior microabscesses, which is recognized as encephalomyelitis type. On the other hand, in encephalomyelitis, abnormal imaging findings other than rim enhancement may be cerebritis, early stage of brain abscess. Moreover, the contrast-enhanced imaging characteristics including nodular, irregular and linear enhancement were not explicitly defined on the reports.

### Conclusions

The CNS melioidosis mostly occurs in adults, and diabetes mellitus is the most common risk factor. Encephalomyelitis and brain abscess are the major types of the disease which fever, unilateral weakness, and cranial nerve deficits are the most prominent presenting features. The CSF profile usually shows mononuclear pleocytosis with high protein and normal glucose level. Contrast-enhanced MRI is the most sensitive imaging study for detecting brain lesions. The rim-enhancing pattern is the most frequent finding, and the brainstem is the most affected location. One-fifth of the patients is fatal. The interpretation from this study may be biased as there were several missing data from the case reports and case series.

## Supporting information

S1 Table Characteristics of included studies and patients. (DOCX)

S2 Table PRISMA-IPD Checklist. (DOCX)
