# Supplementary material for: Central nervous system melioidosis: A systematic review of individual participant data of case reports and case series
Source: PLoS Negl Trop Dis. 2019 Apr 25;13(4):e0007320. doi: 10.1371/journal.pntd.0007320 (PMC6504113; doi:10.1371/journal.pntd.0007320)
Supplement: S1 Table — (DOCX) [file pntd.0007320.s001.docx]

**S1 Table: Characteristics of included studies and patients**

|  | **Reported country** | **Age (years)/ sex^a^** | **Risk** | **Clinical manifestations** | **Type of disease** | **Diagnostic confirmation** | **Outcome** |
| --- | --- | --- | --- | --- | --- | --- | --- |
| Brill et al (1977) [1] | USA | 61/ F | No | Fever, headache, AOC, unilateral weakness, cerebellar sign | Encephalomyelitis | Culture (brain tissue, blood) | Death |
| Beck et al (1984) [2] | USA | 34/ M | No | Fever, headache, CN palsy (III) | Isolated meningitis | Serology | Full recovery |
| Lee et al (1986) [3] | Malaysia | 18/ M | N/A | Fever, headache, AOC, unilateral weakness, CN palsies (VI, VII), neck stiffness | Brain abscess | Culture (brain pus) | Full recovery |
| Pit et al (1988) [4] | Malaysia | 48/ F | N/A | Fever, headache, AOC, unilateral weakness, cranial swelling, neck stiffness | Brain abscess | Culture (brain pus, blood, skull, scalp pus) | Death |
| Yee et al (1988) [5] | Malaysia | 18/ M | No | Fever, headache, AOC, unilateral weakness, CN palsies (VI, VII), neck stiffness | Brain abscess | Culture (brain pus) | Full recovery |
| Pelekanos et al (1989) [6] | Australia | 12/ F | N/A | Fever, headache, AOC, seizures, unilateral weakness, CN palsies (III, VI, VII), respiratory failure | Brain abscess | Culture (skin pus) | Partial recovery |
| Bartley et al (1990) [7] | Australia | 23/ M | No | Fever, AOC, paraplegia | Encephalomyelitis | Culture (blood) | No recovery |
| Visudhiphan et al (1990) [8] | Thailand | 3/ M | N/A | Headache, AOC, seizures, unilateral weakness | Brain abscess | Culture (brain pus) | Partial recovery |
|  |  | 2/ M | N/A | Fever, AOC, seizures, unilateral weakness, CN palsy (VII), neck stiffness | Encephalomyelitis | Culture (CSF, skin pus) | Partial recovery |
|  |  | 8/ M | N/A | AOC, unilateral weakness | Brain abscess | Culture (brain pus) | Partial recovery |
| Kasantikul et al (1992) [9] | Thailand | 51/ F | N/A | Fever, headache, AOC, unilateral weakness, CN palsy (VII) | Brain abscess | Culture (blood) | Death |
|  |  | 9/ M | No | Fever, headache, unilateral weakness, CN palsy (VII) | Brain abscess | Culture (brain pus) | Full recovery |
| Woods et al (1992) [10] | Australia | 39/ F | N/A | Fever, headache, CN palsies (III, IV, VI, VII, IX, X, XI, XII), quadriplegia, neck stiffness, cerebellar sign, respiratory failure | Encephalomyelitis | Culture (skin pus) | Partial recovery |
|  |  | 20/ M | N/A | AOC, CN palsies (III, IV, VI, VII, IX, X, XI), quadriplegia, neck stiffness, respiratory failure | Encephalomyelitis | Culture (blood) | Death |
|  |  | 51/ M | N/A | CN palsies (IX, X, XI), quadriplegia, neck stiffness, respiratory failure | Encephalomyelitis | Culture (blood, sputum) | Partial recovery |
|  |  | 25/ F | N/A | Unilateral weakness, CN palsies (VI, VII, IX, X, XI), neck stiffness | Encephalomyelitis | Culture (CSF, skin pus) | Partial recovery |
| Kong et al (1993) [11] | Singapore | 41/ M | N/A | Fever, AOC, unilateral weakness, neck stiffness | Brain abscess | Culture (brain pus, blood, skin pus) | Partial recovery |
| Chotmongkol et al (1996) [12] | Thailand | 75/ M | N/A | Fever, headache, AOC, neck stiffness | Isolated meningitis | Culture (CSF, blood, urine) | Full recovery |
|  |  | 35/ M | N/A | Fever, headache, neck stiffness | Isolated meningitis | Culture (CSF, blood, splenic pus) | Partial recovery |
| Hiransuthikul et al (1996) [13] | Thailand | 52/ M | N/A | Fever, alteration of consciousness, neck stiffness | Isolated meningitis | Culture (CSF, blood) | Death |
| Wells et al (1996) [14] | Australia | 31/ M | N/A | Fever, headache, unilateral weakness, neck stiffness | Encephalomyelitis | Serology | Death |
| Areekul et al (1997) [15] | Thailand | 18/ M | DM | Fever, headache, neck stiffness, respiratory failure | Isolated meningitis | Culture (blood, sputum) | Death |
| Lath et al (1998) [16] | India | 35/ F | No | Fever, seizures, unilateral weakness, CN palsy (VII) | Brain abscess | Culture (brain pus) | Death |
| Maguire et al (1998) [17] | Australia | 53/ M | No | Fever, headache, unilateral weakness, CN palsy (VI) | Encephalomyelitis | Culture (blood) | Partial recovery |
| Padiglione et al (1998) [18] | Australia | 20/ M | No | Fever, headache, AOC, CN palsies (III, IV, VI, VII, IX, X), neck stiffness, cerebellar sign | Brain abscess | Culture (CSF, blood, sputum, skin pus) | Death |
| Saipan (1998) [19] | Thailand | 4/ M | N/A | Fever, AOC, orbital swelling, neck stiffness | Isolated meningitis | Culture (CSF, blood, eye discharge) | Death |
|  |  | 5 months/ M | N/A | Fever, AOC, seizures | Isolated meningitis | Culture (CSF) | Full recovery |
|  |  | 14/ F | N/A | AOC, seizures, CN palsy (VII) | Encephalomyelitis | Culture (pus from parotid gland) | Partial recovery |
|  |  | 6/ M | N/A | AOC, unilateral weakness, CN palsies (VII, IX, X, XII), neck stiffness | Encephalomyelitis | Culture (pus from parotid gland) | Death |
|  |  | 3/ M | N/A | Fever, seizures, unilateral weakness | Encephalomyelitis | Culture (skin pus) | Partial recovery |
| Halder et al (1998) [20] | Malaysia | 10 days/ M | No | Fever, AOC, seizures | Isolated meningitis | Culture (CSF, blood) | Full recovery |
| Peetermans et al (1999) [21] | Belgium | 66/ M | No | Fever, headache, AOC | Encephalomyelitis | Culture (sputum) | Partial recovery |
| Thummakul et al (1999) [22] | Thailand | 52/ M | No | Fever, headache, AOC, neck stiffness, respiratory failure | Isolated meningitis | Culture (CSF) | Death |
| Currie et al (2000)^b^ [23] | Australia | Age range 20-68  Male 9 cases  Female 3 cases | DM 1, excessive alcohol use 5, chronic lung disease 1, Kava 4 | Fever 11, headache 8, AOC 1, unilateral weakness 6, paraparesis 2, CN palsies (VI 5, VII 8, bulbar 6), neck stiffness 6, cerebellar signs 6, respiratory failure 5 | Encephalomyelitis 9  Isolated meningitis 3 | Culture (brain tissue 1, CSF 1, blood 2, sputum 2, skin pus 1, urine 1)  Serology 3  PCR 1 | Full recovery 3  Partial recovery 4  No recovery 2  Death 3 |
| Edmond et al (2001) [24] | Australia | 11/ M | No | Fever, paraplegia | Encephalomyelitis | Serology | No recovery |
|  |  | 9/ F | No | Fever, CN palsies (VII, IX, X, XII), quadriplegia, respiratory failure | Encephalomyelitis | Culture (sputum) | Partial recovery |
| Haran et al (2001) [25] | Australia | 42/ M | Excessive alcohol use, kava | Fever, paraplegia | Encephalomyelitis | Culture (urine) | No recovery |
| Chadwick et al (2002) [26] | Singapore | 52/ M | DM | AOC, unilateral weakness | Brain abscess | Culture (brain pus, sputum) | Partial recovery |
|  |  | 60/ M | DM, excessive alcohol use | Fever, headache, AOC, seizures | Brain abscess | Serology | Full recovery |
|  |  | 29/ M | DM | Seizures | Brain abscess | Culture (brain pus, subdural pus, blood, skin pus, joint fluid) | Full recovery |
|  |  | 46/ M | No | Unilateral weakness, CN palsy (VII) | Brain abscess | Culture (brain pus) | Partial recovery |
|  |  | 74/ F | No | Fever, headache, seizures | Brain abscess | Culture (blood) | Full recovery |
| Vachvanichsanong et al (2002) [27] | Thailand | 14/ F | No | Fever, seizures | Brain abscess | Culture (pleural fluid) | Full recovery |
| Ganesan et al (2003) [28] | Malaysia | 45/ M | DM | Fever, paraplegia | Isolated extra-axial abscess^c^ | Culture (epidural pus, blood) | Full recovery |
| Bergin et al (2005) [29] | Australia | 69/ F | DM | Fever, headache, AOC | Encephalomyelitis | Culture (brain tissue) | Death |
|  |  | 30/ F | DM, excessive alcohol use | Fever, headache, AOC, seizures | Encephalomyelitis | Culture (brain tissue) | Partial recovery |
| Limmathurotsakul et al (2007) [30] | Thailand | 68/ M | CKD | Fever, headache, AOC, unilateral weakness, CN palsy (VII) | Brain abscess | Culture (brain pus) | Full recovery |
|  |  | 51/ F | yes | Fever, seizures, unilateral weakness | Encephalomyelitis | Culture (epidural pus, blood) | Full recovery |
|  |  | 45/ F | DM | Fever, cranial swelling | Isolated extra-axial abscess | Culture (epidural pus, urine) | N/A |
| Muthusamy et al (2007) [31] | Malaysia | 18/ M | No | Fever, headache, AOC, seizures, unilateral weakness, CN palsies (VI, VII), neck stiffness | Brain abscess | Culture (brain pus) | N/A |
|  |  | 45/ M | DM | Paraplegia | Isolated extra-axial abscess | Culture (epidural pus) | Full recovery |
|  |  | 33/ M | No | Fever, AOC, seizures, neck stiffness | Brain abscess | Culture (brain pus, blood) | Full recovery |
| Kumar et al (2008) [32] | India | 35/ M | N/A | Headache, unilateral weakness | Brain abscess | Culture (brain pus) | Full recovery |
|  |  | 41/ F | N/A | Headache, cranial swelling | Brain abscess | Culture (brain pus) | Full recovery |
|  |  | 40/ M | N/A | Headache, unilateral weakness | Brain abscess | Culture (brain pus) | Full recovery |
| Chlebicki et al (2008) [33] | Singapore | 64/ M | Excessive alcohol use | Fever, AOC, neck stiffness | Isolated meningitis | Culture (CSF, blood) | Partial recovery |
| Falade et al (2008) [34] | USA | 64/ M | DM | Fever, AOC, paraplegia | Isolated extra-axial abscess | Culture (skin pus) | Partial recovery |
| Jusoh et al (2009) [35] | Malaysia | 55/ M | DM | Fever, CN palsies (II, III, IV, VI) | Encephalomyelitis | Culture (blood, eye discharge) | Partial recovery |
| Bommakanti et al (2010) [36] | India | 52/ M | No | Fever, headache, AOC, seizures | Brain abscess | Culture (brain pus, blood) | Full recovery |
| Kuan et al (2010) [37] | Malaysia | 48/ M | DM | Fever, AOC, cranial swelling | Isolated extra-axial abscess | Culture (subdural pus, blood, scalp pus) | Death |
| Periyasamy et al (2011) [38] | Malaysia | 57/ M | DM | Cranial swelling | Isolated extra-axial abscess | Culture (subdural pus) | Full recovery |
| Nandasiri et al (2012) [39] | Sri Lanka | 21/ M | No | Fever, paraplegia | Encephalomyelitis | Culture (pus from muscle) | No recovery |
| Naha et al (2012) [40] | India | 32/ M | DM | Seizures, cranial swelling | Isolated extra-axial abscess | Culture (scalp pus) | Full recovery |
| Cohn et al (2012) [41] | Australia | 14/ F | N/A | Fever, headache, AOC, CN palsies (VII, IX, X, XII), cerebellar sign, respiratory failure | Encephalomyelitis | Culture (brain tissue) | Partial recovery |
| Kung et al (2013) [42] | Taiwan | 45/ M | DM, excessive alcohol use | Fever, AOC, CN palsy (VII), cerebellar sign | Encephalomyelitis | Culture (blood, urine) | Death |
| Vestal et al (2013) [43] | USA | 58/ M | No | Fever, headache, AOC, unilateral weakness, CN palsies (III, XII), neck stiffness | Brain abscess | Culture (brain tissue) | Partial recovery |
| Deuble et al (2013) [44] | Australia | 23/ F | No | Unilateral weakness | Encephalomyelitis | Culture (blood) | No recovery |
|  |  | 43/ F | DM | Fever | Isolated extra-axial abscess | Culture (epidural pus) | Full recovery |
|  |  | 69/ F | DM | Fever, headache | Brain abscess | Culture (brain tissue) | Death |
|  |  | 12/ F | DM | Fever, headache, AOC, unilateral weakness, CN palsy (V), neck stiffness | Encephalomyelitis | Culture (CSF) | No recovery |
|  |  | 56/ M | No | Fever, headache, unilateral weakness, CN palsy (V) | Brain abscess | Culture (sputum) | Death |
|  |  | 41/ M | Excessive alcohol use | Fever, unilateral weakness | Encephalomyelitis | Culture (sputum) | Death |
|  |  | 62/ F | No | Fever, headache, neck stiffness | Isolated meningitis | Culture (CSF) | Full recovery |
|  |  | 43/ M | Excessive alcohol use | Fever, headache, unilateral weakness | Brain abscess | Culture (blood) | No recovery |
|  |  | 14/ F | No | Fever, headache, CN palsies (V, VII, IX, X, XI, XII) | Encephalomyelitis | Culture (brain tissue) | No recovery |
| Kogilavaani et al (2014) [45] | Malaysia | 11/ F | No | Fever, alteration of consciousness, CN palsies (III, IV, VI), unilateral weakness | Isolated extra-axial abscess | Serology | Full recovery |
| Samad et al (2014) [46] | Brunei | 34/ F | N/A | Seizures, respiratory failure | Brain abscess | Culture (CSF, blood) | Partial recovery |
| Arif et al (2015) [47] | UAE | 33/ M | No | AOC, CN palsies (IX, X), quadriparesis, respiratory failure | Brain abscess | Culture (sputum) | Partial recovery |
| Saravu et al (2015) [48] | India | 39/ M | DM, excessive alcohol use | Fever, seizures, unilateral weakness | Isolated extra-axial abscess | Culture (blood) | Partial recovery |
|  |  | 45/ M | N/A | Paraplegia | Brain abscess | Culture (CSF) | No recovery |
| Shetty et al (2015) [49] | India | 55/ M | DM | Fever, seizures, respiratory failure | Encephalomyelitis | Culture (blood, sputum) | Full recovery |
| Vaid et al (2015) [50] | India | 56/ F | DM | Fever, facial swelling | Encephalomyelitis | Culture (blood) | Full recovery |
| Garg et al (2015) [51] | India | 47/ M | DM | Fever, headache | Isolated extra-axial abscess | Culture (epidural pus) | Full recovery |
| Singh et al (2015) [52] | India | 51/ M | N/A | Fever, AOC, neck stiffness, respiratory failure | Isolated meningitis | Culture (CSF, blood) | Full recovery |
| Hesstvedt et al (2015) [53] | Norway | 26/ F | No | Fever, headache, seizures, unilateral weakness | Brain abscess | Culture (brain pus) | Full recovery |
| Hsu et al (2016) [54] | Australia | 52/ M | N/A | Fever, headache | Isolated extra-axial abscess | Culture (epidural pus) | Partial recovery |
| Liang et al (2016) [55] | Taiwan | 30/ M | N/A | Fever, AOC, seizures, unilateral weakness | Brain abscess | Culture (brain pus, CSF, blood) | Partial recovery |
| Madi et al (2016) [56] | India | 54/ M | No | Fever, headache, seizures, cranial swelling | Isolated extra-axial abscess | Culture (scalp pus) | Full recovery |
| White et al (2016) [57] | Australia | 14/ M | N/A | Headache, AOC, unilateral weakness, CN palsies (VI, VII), cerebellar sign | Brain abscess | Culture (brain pus) | Partial recovery |
| Ng et al (2016) [58] | Phillippines | 65/ M | DM | Headache, seizures | Isolated meningitis | Culture (CSF) | Death |
| Andersen et al (2016) [59] | Australia | 4/ M | N/A | Fever, AOC, paraplegia | Encephalomyelitis | Serology | Partial recovery |
| Fong et al (2017) [60] | Malaysia | 46/ F | DM | Fever, headache, AOC, cranial swelling | Brain abscess | Culture (scalp pus) | Full recovery |
|  |  | 16/ M | N/A | Fever, seizures | Brain abscess | Culture (blood) | Full recovery |
|  |  | 67/ F | DM | Fever, AOC, unilateral weakness | Brain abscess | Culture (blood) | Full recovery |
| Katanami et al (2017) [61] | Japan | 40/ M | No | Fever, respiratory failure | Encephalomyelitis | Culture (blood) | Death |
| Prasanna Kumar et al (2017) [62] | India | 10 months/ F | N/A | Fever | Brain abscess | Culture (skin pus, bone) | Full recovery |
| Loh et al (2017) [63] | Australia | 35/ M | DM | Fever, AOC, facial swelling, respiratory failure | Brain abscess | Culture (blood, sputum, ear discharge) | Full recovery |
| Zhan et al (2017) [64] | China | 31/ M | N/A | Fever, headache | Encephalomyelitis | Culture (blood) | No recovery |
|  |  | 56/ M | DM | Chest pain | Encephalomyelitis | Culture (blood) | No recovery |
|  |  | 65/ M | DM | Fever, unilateral weakness | Encephalomyelitis | Culture (brain pus) | No recovery |
|  |  | 9/ M | N/A | Fever | Isolated meningitis | Culture (blood) | Partial recovery |
|  |  | 60/ M | DM | Headache | Isolated extra-axial abscess | Culture (blood) | No recovery |
|  |  | 66/ M | DM | Fever, headache | Isolated meningitis | Culture (blood) | N/A |
| Ekka et al (2017) [65] | India | 11/ F | No | Fever, AOC, quadriplegia, neck stiffness | Encephalomyelitis | Culture (brain tissue) | Partial recovery |
| Prasad et al (2017) [66] | India | 41/ M | No | Fever, cranial swelling | Isolated extra-axial abscess | Culture (scalp pus) | Full recovery |
| Toh et al (2017) [67] | Malaysia | 15/ F | DM | Fever, seizures | Brain abscess | Culture (skin pus) | Full recovery |
| Wongwandee et al (2017) [68] | Thailand | 55/ M | DM, excessive alcohol use | Seizures, unilateral weakness | Brain abscess | Culture (brain tissue) | Partial recovery |
| Yong et al (2017) [69] | Malaysia | 18/ M | No | Fever, headache, alteration of consciousness, unilateral weakness, neck stiffness | Brain abscess | Culture (blood) | Full recovery |
| Chen et al (2018) [70] | Taiwan | 65/ F | No | Fever, headache, AOC | Isolated meningitis | Culture (CSF) | Full recovery |

N/A, not available; M, male; F, female; DM, diabetes mellitus; CN, cranial nerve; AOC, alteration of consciousness; CSF, cerebrospinal fluid; PCR, polymerase chain reaction

^a^ Only the number of patients with central nervous system melioidosis is provided. The publication may report additional cases.

^b^ Only aggregate data are available.

^c^ Isolated extra-axial collection is defined as subdural empyema or epidural abscess with the absence of encephalomyelitis and brain abscess.

**References**

1. Brill DR, Shoop JD. Sensitivity of radionuclide isotope brain scan in cerebral melioidosis: case report. J Nucl Med. 1977;18: 987-989.
2. Beck RW, Janssen RS, Smiley ML, Schatz NJ, Savino PJ, Rubin DH. Melioidosis and bilateral third-nerve palsies. Neurology. 1984; 34: 105-107.
3. Lee M, Chua C. Brain abscess due to Pseudomonas pseudomallei. Aust N Z J Med. 1986; 16: 75-77.
4. Pit S, Chea FK, Jamal F. Melioidosis with brain abscess. Postgrad Med J. 1988; 64: 140-142.
5. Yee KC, Lee MK, Chua CT, Puthucheary SD. Melioidosis, the great mimicker: a report of 10 cases from Malaysia. J Trop Med Hyg. 1988; 91: 249-254.
6. Pelekanos JT, Appleton DB. Melioidosis with multiple cerebral abscesses. Pediatr Neurol 1989; 5: 48-52.
7. Bartley PP, Pender MP, Woods ML, Walker D, Douglas JA, Allworth AM, et al. Spinal cord disease due to melioidosis. Trans R Soc Trop Med Hyg. 1999; 93: 175-176.
8. Visudhiphan P, Chiemchanya S, Dheandhanoo D. Central nervous system melioidosis in children. Pediatr Infect Dis J. 1990; 9: 658-661.
9. Kasantikul V, Lerdlum S, Suwanwela N. Cerebral abscesses due to Pseudomonas pseudomallei. J Med Assoc Thai. 1992; 75: 536-541.
10. Woods ML 2nd, Currie BJ, Howard DM, Tierney A, Watson A, Anstey NM, et al. Neurological melioidosis: seven cases from the Northern Territory of Australia. Clin Infect Dis. 1992; 15: 163-169.
11. Kong HL, Ong BK, Lee TK, Cheah JS. Melioidosis of the brain presenting with a stroke syndrome. Aust N Z J Med. 1993; 23: 413-414.
12. Chotmongkol V, Sukeepaisarncharoen W. Burkholder meningitis. J Med Assoc Thai. 1996; 79: 263-266.
13. Hiransuthikul N, Tantawichien T. Melioidosis with erythroderma and meningitis: a case report. Chula Med J. 1996; 40: 837-843.
14. Wells R, McCormack J, Lavercombe P, Tannenberg A. Melioidosis causing encephalomyelitis. Aust N Z J Med. 1996; 26: 567.
15. Areekul S, Vongsthongsri U, Wattanarungson C, Cheattanadee S, Wilairatana P. Septicemic melioidosis and meningitis: a case report. Siriraj Hosp Gaz. 1997; 49: 1084-1087.
16. Lath R, Rajshekhar V, George V. Brain abscess as the presenting feature of melioidosis. Br J Neurosurg. 1998; 12: 170-172.
17. Maguire GP, Flavell HD, Burrow JN, Currie BJ. Relapsing neurological melioidosis from the top end of the Northern Territory. Aust N Z J Med. 1998; 28: 219-220.
18. Padiglione A, Ferris N, Fuller A, Spelman D. Brain abscesses caused by Burkholderia pseudomallei. J Infect. 1998; 36: 335-337.
19. Saipan P. Neurological manifestations of melioidosis in children. Southeast Asian J Trop Med Public Health. 1998; 29: 856-859.
20. Halder D, Zainal N, Wah CM, Haq JA. Neonatal meningitis and septicaemia caused by Burkholderia pseudomallei. Ann Trop Paediatr. 1998; 18: 161-164.
21. Peetermans WE, Van Wijngaerden E, Van Eldere J, Verhaegen J. Melioidosis brain and lung abscess after travel to Sri Lanka. Clin Infect Dis. 1999; 28: 921-922.
22. Thummakul T, Wilde H, Tantawichien T. Melioidosis, an environmental and occupational hazard in Thailand. Mil Med. 1999; 164: 658-662.
23. Currie BJ, Fisher DA, Howard DM, Burrow JN. Neurological melioidosis. Acta Trop. 2000; 74: 145-151.
24. Edmond KM, Bauert P, Currie BJ. Paediatric melioidosis in the Northern Territory of Australia: an expanding clinical spectrum. J Paediatr Child Health. 2001; 37: 337-341.
25. Haran MJ, Jenney AW, Keenan RJ, Flavell HD, Anstey NM, Currie BJ. Paraplegia secondary to Burkholderia pseudomallei myelitis: a case report. Arch Phys Med Rehabil. 2001; 82: 1630-1632.
26. Chadwick DR, Ang B, Sitoh YY, Lee CC. Cerebral melioidosis in Singapore: a review of five cases. Trans R Soc Trop Med Hyg. 2002; 96: 72-76.
27. Vachvanichsanong P, Dissaneewate P, Prukprasert P, Wongchanchailert M, Thongmak S, Janjindamai S, et al. Melioidosis and brain abscess in a girl with systemic lupus erythematosus. Infect Dis Clin Pract (Baltim Md). 2002; 11: 211-213.
28. Ganesan D, Puthucheary SD, Waran V. Melioidosis presenting as spinal epidural abscess. Br J Neurosurg. 2003; 17: 568-571.
29. Bergin P, Boyes L, Sage M. Cerebral melioidosis. Australas Radiol. 2005; 49: 79-83.
30. Limmathurotsakul D, Chaowagul W, Wongsrikaew P, Narmwong A, Day NP, Peacock SJ. Variable presentation of neurological melioidosis in Northeast Thailand. Am J Trop Med Hyg. 2007; 77: 118-120.
31. Muthusamy KA, Waran V, Puthucheary SD. Spectra of central nervous system melioidosis. J Clin Neurosci. 2007; 14: 1213-1215.
32. Kumar GS, Raj PM, Chacko G, Lalitha MK, Chacko AG, Rajshekhar V. Cranial melioidosis presenting as a mass lesion or osteomyelitis. J Neurosurg. 2008; 108: 243-247.
33. Chlebicki MP, Kurup A, Sin YK. Burkholderia pseudomallei meningitis following inadequate treatment of melioidotic mycotic aneurysm. Singapore Med J. 2008; 49: e219-e221.
34. Falade OO, Antonarakis ES, Kaul DR, Saint S, Murphy PA. Clinical problem-solving. Beware of first impressions. N Engl J Med. 2008; 359: 628-634.
35. Jusoh S, Shaharuddin B, Ismail S. Successfully treated rare presentation of orbital melioidosis. Int J Ophthalmol. 2009; 2: 90-92.
36. Bommakanti K, Ankathi P, Uma P, Malladi S, Laxmi V. Cerebral abscess and calvarial osteomyelitis due to Burkholderia pseudomallei. Neurol India. 2010; 58: 801-802.
37. Kuan YC, How SH, Ng TH, Fauzi AR. The man with the boggy head: cranial melioidosis. Singapore Med J. 2010; 51: e43-e45.
38. Periyasamy P, Kadir A, Nyiu C. PP-012 Cotrimoxazole resistant Burkholderia pseudomallei in neurological melioidosis [abstract]. Int J Infect Dis. 2011; 15: S49.
39. Nandasiri S, Wimalaratna H, Manjula M, Corea E. Transverse myelitis secondary to melioidosis: a case report. BMC Infect Dis. 2012; 12: 232.
40. Naha K, Dasari S, Kusugodlu R, Prabhu M. Cranial melioidosis with extradural extension after a fall in the bathroom. Australas Med J. 2012; 5: 455-458.
41. Cohn A, Norton R, Walsh M, Nourse C. Neurologic melioidosis in a child: unique clinical features and challenges of serologic diagnosis. Pediatr Infect Dis J. 2012; 31: 1197-1198.
42. Kung CT, Li CJ, Ko SF, Lee CH. A melioidosis patient presenting with brainstem signs in the emergency department. J Emerg Med. 2013; 44: e9-e12.
43. Vestal ML, Wong EB, Milner DA, Jr., Gormley WB, Dunn IF. Cerebral melioidosis for the first time in the western hemisphere. J Neurosurg. 2013; 119: 1591-1595.
44. Deuble M, Aquilina C, Norton R. Neurologic melioidosis. Am J Trop Med Hyg. 2013; 89: 535-539.
45. Kogilavaani J, Shatriah I, Regunath K, Helmy A. Bilateral orbital abscesses with subdural empyema and cavernous sinus thrombosis due to melioidosis in a child. Asian Pac J Trop Dis. 2014; 4: S851–S853.
46. Samad I, Wang MC, Chong VH. Intracerebral coinfection with Burkholderia pseudomallei and Cryptococcus neoformans in a patient with systemic lupus erythematosus. Southeast Asian J Trop Med Public Health. 2014; 45: 352-356.
47. Arif MA, Abid MH, Renganathan R, Siddiqui KA. Central and peripheral nervous system involvement in neuromelioidosis. BMJ Case Rep. 2015 Oct 22. doi: 10.1136/bcr-2015-211001.
48. Saravu K, Kadavigere R, Shastry AB, Pai R, Mukhopadhyay C. Neurologic melioidosis presented as encephalomyelitis and subdural collection in two male labourers in India. J Infect Dev Ctries. 2015; 9: 1289-1293.
49. Shetty HS, Mallela AR, Shastry BA, Acharya V. Parietal bone osteomyelitis in melioidosis. BMJ Case Rep. 2015 Feb 27. doi: 10.1136/bcr-2014-208612.
50. Vaid T, Rao K, Hande HM. An intriguing case of locked jaw secondary to melioidosis. BMJ Case Rep. 2015 Dec 1. doi: 10.1136/bcr-2015-213060.
51. Garg G, Chawla N, Chawla K, Khosla P, Jain S. Atypical Presentations of Melioidosis in North India: Report of Two Cases. J Assoc Physicians India. 2015; 63: 82-83.
52. Singh A, Grover N, Gupta S, Bhatt P, Sahni A. Disseminated melioidosis: a case report. Rev Med Microbiol. 2015; 26: 116-118.
53. Hesstvedt L, Reikvam DH, Dunlop O. Neurological melioidosis in Norway presenting with a cerebral abscess. IDCases. 2015; 2: 16-18.
54. Hsu CC, Singh D, Kwan G, Deuble M, Aquilina C, Korah I, et al. Neuromelioidosis: Craniospinal MRI Findings in Burkholderia pseudomallei Infection. J Neuroimaging. 2016; 26: 75-82.
55. Liang CC, Chen SY, Chen TY, Chen ST. Central Nervous System Melioidosis Mimics Malignancy: A Case Report and Literature Review. World Neurosurg. 2016; 89: e19-e23.
56. Madi D, Rai SP, Vidyalakshmi K, Chowta KN. Neurological melioidosis presenting as intracranial abscess. Indian J Pathol Microbiol. 2016; 59: 417-419.
57. White ME, Hunt J, Connell C, Langdon K. Paediatric neurological melioidosis: a rehabilitation case report. Rural Remote Health. 2016; 16: 3702.
58. Ng JPO, Amsua LC, Licup G. Sporadic neurologic melioidosis presenting as primary meningitis in a 65 year old diabetic male: a case report [abstract]. Shock. 2016; 46: 49.
59. Andersen EW, Mackay MT, Ryan MM. Neurologic Melioidosis: Case Report of a Rare Cause of Acute Flaccid Paralysis. J Pediatr. 2016; 170: 319-321.
60. Fong SL, ShyanWong J, Tan AH, Low SC, Tan CT. Neurological melioidosis in East Malaysia: Case series and review of the literature. Neurol Asia. 2017; 22: 25-32.
61. Katanami Y, Kutsuna S, Horino A, Hashimoto T, Mutoh Y, Yamamoto K, et al. A fatal case of melioidosis with pancytopenia in a traveler from Indonesia. J Infect Chemother. 2017; 23: 241-244.
62. Prasanna Kumar M, Krishnamurthy S, Venkateswaran V, Mahadevan S, Lalitha M, Sistla S, et al. Brainstem micro-abscesses caused by Burkholderia pseudomallei in a 10-month-old infant: a case report. Paediatr Int Child Health. 2017; 37: 230-232.
63. Loh TL, Latis S, Crossland G, Patel H. Disseminated melioidosis in the head and neck. BMJ Case Rep. 2017 Jan 17. doi: 10.1136/bcr-2016-218606.
64. Zhan Y, Wu Y, Li Q, Yu A. Neuromelioidosis: a series of seven cases in Hainan province, China. J Int Med Res. 2017; 45: 856-867.
65. Ekka AS, Mohideen M, Kesavan S. Neuromelioidosis Masquerading as Acute Demyelinating Encephalomyelitis. Indian Pediatr. 2017; 54: 1054-1055.
66. Prasad GL, Nair RP, Menon GR. Intracranial melioidosis: First report in a human immunodeficiency virus positive individual manifesting as cranial osteomyelitis. Neurol India. 2017; 65: 1423-1426.
67. Toh T, Chong H. Brain abscess-not to forget melioidosis [abstract]. J Neurol Sci. 2017; 381: 1013.
68. Wongwandee M, Kiatsoontorn K, Linasmita P. Central Nervous System Melioidosis, the Mimic of Cerebral Tuberculosis. J Med Assoc Thai. 2017; 100: S208-S211.
69. Yong SS, Hassan SA, Wong PS, Yoong KY. A Rare Presentation of Central Nervous System Melioidosis. OAlib. 2017 Jan 20. doi: 10.4236/oalib.1103346.
70. Chen GB, Tuan SH, Chen LH, Lin WS. Neurological melioidosis (Burkholderia pseudomallei) in a chronic psychotic patient treated with antipsychotics: A case report. Medicine (Baltimore). 2018; 97: e11110.
